# Prevalence of Tongue Cleaning Using a Toothbrush: A Questionnaire Survey in Fukui Prefecture, Japan

**DOI:** 10.1155/2019/6320261

**Published:** 2019-11-04

**Authors:** Shinpei Matsuda, Takehisa Saito, Hisato Yoshida, Hitoshi Yoshimura, Kazuo Sano

**Affiliations:** ^1^Department of Dentistry and Oral Surgery, Unit of Sensory and Locomotor Medicine, Division of Medicine, Faculty of Medical Sciences, University of Fukui, Fukui, Japan; ^2^Department of Otorhinolaryngology, Head and Neck Surgery, Unit of Sensory and Locomotor Medicine, Division of Medicine, Faculty of Medical Sciences, University of Fukui, Fukui, Japan; ^3^Oral Care Support Center, Fukui Dental Association, Japan

## Abstract

**Objective:**

The aim of this study was to investigate the tongue cleaning habits using toothbrushes among outpatients of the University of Fukui Hospital and a private hospital in Fukui Prefecture.

**Methods:**

We administered a questionnaire survey to volunteers detailing tongue cleaning habits using toothbrushes. The content of the questions in this survey were as follows: gender, age, frequency of tongue cleaning, portion of tongue cleaning, and purposes of tongue cleaning.

**Results:**

We had 1,014 volunteers of various ages participating in this study. Regarding the frequency of tongue cleaning, 187 (18.4%) of all participants replied, “Every day”, and 346 (34.1%) replied, “Sometimes”. Regarding tongue cleaning of the 533 participants with active tongue cleaning habits, 242 (45.4%) participants replied, “The center of the dorsum of the tongue”, and 274 (51.4%) replied, “The entire tongue”. When analyzing the purpose of tongue cleaning, 346 (64.9%) participants replied, “To remove the tongue stain”, 192 (36.0%) participants replied, “To remove the tongue coating”, and 240 (45.0%) participants replied, “To manage halitosis”.

**Conclusions:**

This study clarified that a wide range of age groups in the nonhospitalized general public practiced tongue cleaning habits using a toothbrush for various purposes.

## 1. Introduction

Oral care brings various benefits. In 1999, Yoneyama et al. reported that oral care could decrease the risk of pneumonia in the institutionalized elderly [1]. In institutionalized elderly, the importance of oral care, including tongue cleaning has received attention, and several studies have reported on the topic. Izumi et al. reported that oral care with tongue cleaning is important for preventing aspiration pneumonia, because it could improve coughing ability [2]. Recently, the relationship between tongue coating and food type were clarified, and its result suggested that tongue cleaning should be performed with consideration for the food type [3].

In hospitalized patients, infectious complications, such as ventilator-associated pneumonia (VAP) and surgical site infection, sometimes lead to critical conditions [4–6]. Some studies have suggested that oral care, including tongue cleaning, could reduce those complications, and that oral care improving the quality of patient care was economically viable [4–6]. Also, Tajima et al. reported that tongue cleaning was also useful for elders fed with a feeding tube because it could decrease the number of microbes on the tongue surface [7].

As the aging of the population progresses in Japan, the importance of oral care is widely known not only to institutionalized elderly or hospitalized patients, but also to the nonhospitalized general public. With the increasing interest in oral care, attention has also been focused on tongue cleaning. Tongue cleaning has been practiced for centuries all over the world with tongue scrapers made from various materials, such as silver, gold, copper, tin, brass, and plastic [8]. Tongue cleaning is usually performed to remove tongue coating, and it has been considered to contribute to oral bacterial control and halitosis management [9–13]. Halitosis is not usually associated with acute oral or systemic infections, but it may be associated with chronic periodontal disease or the chronic action of bacteria in tongue coating [14, 15]. In addition, a recent study reported that continuous tongue brushing improved subjective taste, such as sweet, salty, sour, and bitter [16, 17]. Seerangaiyan et al. suggested that tongue cleaning could increase the salt taste intensity and help individuals adhere to the World Health Organization recommendations on dietary salt intake [17]. Furthermore, tongue cleaning might improve gingival inflammation and digestive power [18, 19]. In contrast to those studies, one of our previous studies using confocal laser scanning microscopy in combination with a filter-paper disc method suggested that excessive tongue cleaning might lead to a decreased number of both fungiform papillae and taste buds associated with taste sensation [20]. Based on the results of these studies, we considered that moderate or mild mechanical tongue cleaning may be acceptable and yield some positive effects, but excessive tongue cleaning may affect taste sensation. The chorda tympani nerve innervates the taste buds of the fungiform papillae in the anterior two-thirds of the tongue [21]. Also, the distribution of fungiform papillae innervated by the chorda tympani nerve is substantial in the tip and midlateral regions of the tongue [22, 23]. Therefore, we considered that brushing the entire tongue, including the midlateral and tip regions, might affect the fungiform papillae associated with taste sensation [20].

Recently, many specific tongue cleaning tools have been produced and sold [24, 25]. On the other hand, a recent study on tongue cleaning reported that a toothbrush was the most common tongue cleaning tool [26]. However, most studies about tongue cleaning do not include the toothbrush tongue cleaning practice despite of the difference in mechanical cleaning force applied to the tongue. Additionally, there have been no reports about portion and purposes of the tongue cleaned using a toothbrush. Therefore, we considered that it was important to investigate the actual prevalence, portion, and purposes of tongue cleaning using a toothbrush.

Medical professionals should know the actual conditions of tongue cleaning using a toothbrush. Then, medical professionals should disseminate and give medical advice about oral care based on this information. However, there has been no large-scale survey about the effects of tongue cleaning using toothbrushes or information about the cleaned portion of the tongue. The aim of this study was to investigate the habits of tongue cleaning using toothbrushes among nonhospitalized people in Fukui Prefecture, Japan.

## 2. Materials and Methods

### 2.1. Ethical Approval

This study was an observational study, and approved by the Institutional Research Board (Ethics Committee of University of Fukui, Faculty of Medical Sciences; No. 20150088).

### 2.2. Participants

A questionnaire survey of tongue cleaning using a toothbrush was given to volunteers of various ages between 2015 and 2016. The participants were outpatients of the Department of Otorhinolaryngology, Head and Neck Surgery of University of Fukui Hospital and a private hospital in Fukui Prefecture, Japan. In addition, to eliminate bias, this questionnaire survey was not conducted by dental professionals, including dentists and oral hygienists, or in places related to dental treatment and oral surgery. To obtain appropriate answers for questionnaires, only populations aged 15 or over participated in this study. Participants with incomplete responses to the questionnaire were excluded.

### 2.3. Questionnaires

The content of the questions in this survey was as follows: (1) gender, (2) age, (3) frequency of tongue cleaning, (4) portion of tongue cleaning, and (5) purposes of the tongue cleaning. The ages were divided into “10s”, “20s”, “30s”, “40s”, “50s”, “60s”, “70s”, and “80s and over”. The frequency of tongue cleaning was divided into “Every day”, “Sometimes”, and “Never”. In this questionnaire survey, “Never” meant that participants had no experience with the tongue cleaning using a toothbrush for tongue cleaning. The portion of the tongue cleaning detailed the following three cases: “The center of the dorsum of the tongue”, “The entire tongue”, and “The others”. The following three purposes of tongue cleaning were detailed: “To remove tongue stain”, “To remove tongue coating”, and “To manage halitosis”. Also, multiple answers were permitted only for the question about purposes of the tongue cleaning.

### 2.4. Statistical Analyses

The relationships between frequency of tongue cleaning and each age group, between portion of tongue cleaning and gender, and between purposes of tongue cleaning and gender were analyzed statistically. In frequency analysis, the male and female participant groups were analyzed separately. Statistical analyses were performed using the IBM SPSS Statistics version 25 statistical software (IBM Japan Ltd., Tokyo, Japan). A chi-squared test was used to assess the statistically significant relationship. The value of *p* < 0.05 was considered statistically significant.

## 3. Results

**Figure 1:**
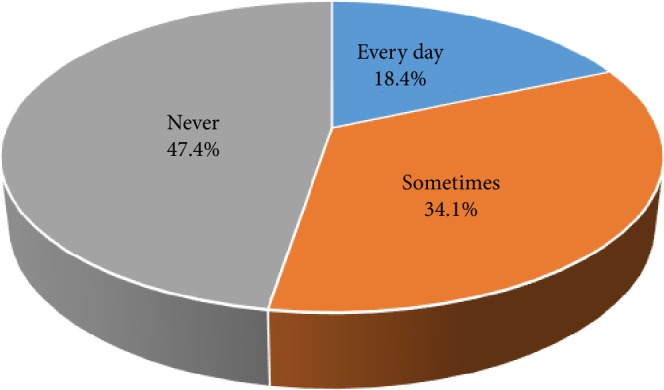
Frequency of tongue cleaning using a toothbrush.

**Figure 2:**
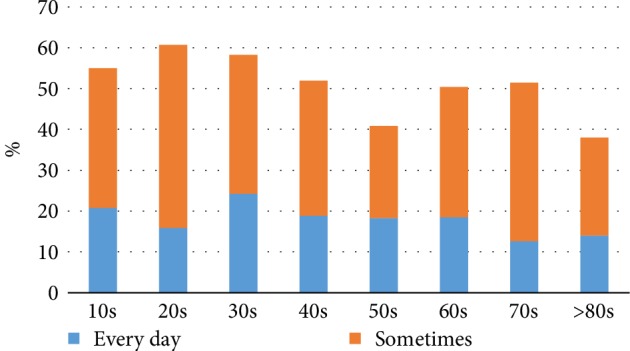
Percentage of participants who had tongue cleaning habits.

**Figure 3:**
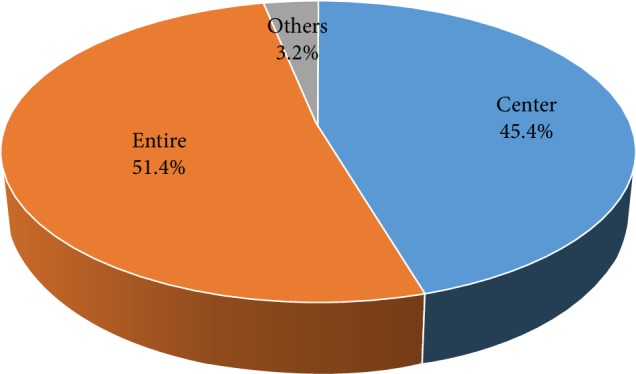
Portion of tongue cleaning using a toothbrush.

**Figure 4:**
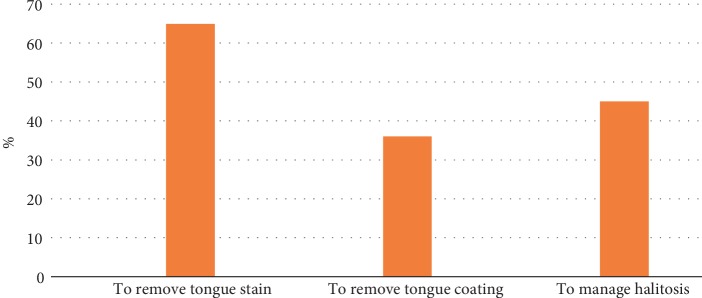
Purpose of tongue cleaning using a toothbrush.

There were 1,014 participants of various ages who completed our questionnaires for this study. The participants consisted of 339 males (33.4%) and 675 females (66.6%). The mean age and standard deviation of those participants was 42.7 ± 21.8 years. The youngest subject was 15 years old, and the oldest subject was 92 years old. The group of “10s” (231 participants, 22.8%) was the largest group, and the group of “80s and over” (50 participants, 4.9%) was the smallest group. The average number and standard deviation of each age group was 126.8 ± 53.7. Regarding the frequency of tongue cleaning, 187 (18.4%) of all participants replied, “Every day”, 346 (34.1%) replied “Sometimes”, and 481 (47.4%) replied “Never” (Table 1, Figure 1). Forty-six (13.6%) male participants replied, “Every day”, 101 (29.8%) replied “Sometimes”, and 192 (56.6%) replied, “Never”. One hundred forty-one (20.9%) female participants replied, “Every day”, 245 (36.3%) replied, “Sometimes”, and 289 (42.8%) replied “Never”. When considering both aspects of the frequency of tongue cleaning and age groups, the highest percentage of participants that replied “Every day” was the 30s group (24.3%), and the lowest was the 70s group (12.6%). In regard to male participants, the highest percentage of respondents that replied, “Every day” was the 60s group (25.0%), and the lowest was the over 80s group (0%). In female participants, the highest percentage of respondents that answered “Every day” was the 30s group (28.8%), and the lowest was the 60s group (13.9%). When summing the percentages of participants who replied, “Every day” and “Sometimes”, namely, percentage of participants who practiced tongue cleaning habits regardless of the frequency, the highest was the 20s group (60.7%), and the lowest was the over 80s group (38.0%) (Figure 2). There was no statistically significant relationship between the frequency of tongue cleaning and age groups in neither male nor female participants (*p* = 0.083 vs. *p* = 0.077, chi-squared tests). In regard to the portion of tongue cleaning, 242 (45.4%) of 533 participants with tongue cleaning habits replied, “The center of the dorsum of the tongue”, and 274 (51.4%) replied, “The entire tongue” (Table 2, Figure 3). There was no statistically significant relationship between portion of tongue cleaning and gender (*p* = 0.564, chi-squared tests). In regard to the purposes of tongue cleaning, 346 (64.9%) of 533 participants replied “To remove the tongue stain,” 192 (36.0%) of them replied “To remove the tongue coating”, and 240 (45.0%) of them replied “To manage halitosis” (Table 3, Figure 4). The analysis of gender differences in the purpose of tongue cleaning based on the sum of participants that replied “Everyday” and “Sometimes” showed as follows: 95 (64.6%) of 147 male participants and 251 (65.0%) of 386 female participants replied “To remove the tongue stain”, 54 (36.7%) male participants and 138 (35.8%) female participants replied “To remove the tongue coating”, and 64 (43.5%) male participants and 176 (45.6%) female participants replied “To manage halitosis”. There was no statistically significant relationship between purposes of tongue cleaning and gender (*p* = 0.944, chi-squared tests).

## 4. Discussion

This study investigated the prevalence of tongue cleaning using a toothbrush through a relatively large-scale questionnaire survey administered to the nonhospitalized general public in Fukui Prefecture, Japan. Although the cleaning frequency was different among age groups, the results of this questionnaire survey clarified that 533 (52.6%) of 1,014 participants had tongue cleaning habits using toothbrushes. There was no statistically significant relationship between the frequency of tongue cleaning and age groups in both male and female participants. These results suggested that a wide range of age groups of the general public have had a great interest in tongue cleaning, indicating that the importance of oral care is widespread not only in medical professionals, but also the general public. In regard to the portion of tongue cleaning, the number of participants that replied, “The entire tongue” was slightly higher than the number of participants that replied, “The center of the dorsum of the tongue”. Also, there was no statistically significant relationship between portion of tongue cleaning and gender. In regard to the purposes of tongue cleaning, the response “To remove the tongue stain” was most common. Interestingly, when analyzing the various percentages for the purposes of tongue cleaning in relation to the participants who had the tongue cleaning habits, regardless of the frequency, we observed close values between male and female participants for all three purposes, and there was no statistically significant relationship between purposes of tongue cleaning and gender. That result indicated that gender did not play a role in tongue cleaning purpose. However, it should be noted that multiple answers were permitted only for the question about purposes of the tongue cleaning. This study suggested that the general public cared about tongue cleanliness and halitosis, and tongue cleaning using a toothbrush was performed in several different ways for different purposes.

Kishi et al. conducted a questionnaire survey about tongue cleaning habits for 479 participants and reported that 37.0% of all participants replied that they practiced tongue cleaning [26]. Although it is widely known that there are special tools for brushing tongues, a toothbrush (81.4%) was the most common tongue cleaning tool [26]. However, there were no questions about the portion of the tongue cleaned by cleaning tools in their study [26]. In this study, a questionnaire survey associated with tongue cleaning using a toothbrush was conducted based on that report. Their survey was conducted at two public health centers that held monthly meetings for health promotion and at one dental hospital [26]. On the other hand, our survey was neither conducted by dental professionals, including dentists and oral hygienists, nor in places related to dental treatment and oral surgery for elimination of bias. Thus, we considered that the results of this questionnaire survey were reliable and beneficial. Because our questionnaires needed to be as simple as possible, we selected five questions, involving gender, age, frequency, portion, and purposes of the tongue cleaning, to clarify the summary of tongue cleaning habits.

The effects of mechanical tongue cleaning were examined in some basic bacteriological studies. Matsui et al. reported about a study using a disposable tongue cleaner equipped with a cleaner head composed of a urethane sponge and their study suggested that tongue cleaning reduced the amount of bacteria on the tongue [9]. Some literature has reported on the effect of tooth brushing accompanied with tongue cleaning compared to tooth brushing only, and the former had a significant effect on the reduction of halitosis and tongue coating [27, 28]. Furthermore, Bordas et al. concluded that mechanical tongue cleaning with or without chemical intervention, such as mouthwash, could reduce bacterial load on the tongue [28].

Mechanical tongue cleaning may have positive effects on oral bacterial load, halitosis management, and subjective taste [9–13, 16, 17]. In contrast to those reports, one of our previous studies suggested that excessive mechanical tongue cleaning might lead to damage of the gustatory receptors, and progression of this damage might have an association with the decreased number of taste buds that exist on the surface of the fungiform papillae [20]. Kullaa-Mikkonen et al. reported that fungiform papillae and taste buds are distributed over the dorsum of the tongue and that they are more common at the tip and on the edge than in the middle of the tongue [22]. Additionally, it is important that the tongue coating, including desquamated epithelial cells, food debris, bacteria, and salivary proteins, was usually found in the mid-distal part of the dorsum of the tongue [15, 29]. The results of these studies suggested that the tongue cleaning should not be performed at the tip and on the edge of the tongue for the purpose of tongue coating removal, although moderate cleaning of the center of the tongue is acceptable. Medical professionals should keep in mind that the oral cavity structures might be easily damaged, and excessive mechanical tongue cleaning may affect those delicate and sensitive anatomical structures, such as fungiform papillae and taste buds.

In this study, we investigated the prevalence of tongue cleaning using only a toothbrush. The limitations of this study were that this was a questionnaire survey and that there is no additional information about the clinical oral environment. Furthermore, to simplify the survey, we chose a selection-type questionnaire in items of frequency, portion, and purposes of the tongue cleaning. In addition, we should consider that there is dispersion in the number of the participants in both aspects of gender and ages in this study. We need to consider the possibility that the results in this study were influenced by characteristics of the Japanese culture and the tendency of Japanese individuals to prefer cleanliness.

With the increasing interest in oral care, prevalence of tongue cleaning and the market of cleaning tools will expand. Rickenbacher et al. reported that acceptance of the use of a tongue vacuum cleaner among children was higher than acceptance with a child's manual toothbrush [25]. However, there are few reports discussing the differences between tongue cleaning using a toothbrush and other mechanical tongue cleaning methods. Also, there are many types of toothbrushes depending on form and hardness. Therefore, we considered that the research on tongue brushing is inadequate at this time. Based on the results obtained from this research, further studies associated with tongue management methods, such as cleaning tools, frequency, degree, and teaching and assistance methods, should be carried out. These results will provide the general public with valuable information on suitable tongue management methods in the future.

## 5. Conclusions

This study found that a wide range of age groups in the nonhospitalized general public performed tongue cleaning using a toothbrush for various purposes. Further studies associated with tongue management methods will provide the general public with valuable tongue cleaning information in the future.

## Figures and Tables

**Table 1:** Frequency of tongue cleaning using a toothbrush.

Frequency	Gender	Every day		Sometimes		Never		Total
Age		*n* (%)		*n* (%)		*n* (%)		
15–19		48 (20.8)		79 (34.2)		104 (45.0)		231 (22.8)
	Male		6 (11.1)		16 (29.6)		32 (59.3)	
	Female		42 (23.7)		63 (35.6)		72 (40.7)	
20–29		23 (15.9)		65 (44.8)		57 (39.3)		145 (14.3)
	Male		10 (13.5)		30 (40.5)		34 (45.9)	
	Female		13 (18.3)		35 (49.3)		23 (32.4)	
30–39		25 (24.3)		35 (34.0)		43 (41.7)		103 (10.2)
	Male		4 (13.3)		8 (26.7)		18 (60.0)	
	Female		21 (28.8)		27 (37.0)		25 (34.2)	
40–49		29 (18.8)		51 (33.1)		74 (48.1)		154 (15.2)
	Male		8 (19.5)		11 (26.8)		22 (53.7)	
	Female		21 (18.6)		40 (35.4)		52 (46.0)	
50–59		17 (18.3)		21 (22.6)		55 (59.1)		93 (9.2)
	Male		1 (3.1)		7 (21.9)		24 (75.0)	
	Female		16 (26.2)		14 (23.0)		31 (50.8)	
60–69		25 (18.5)		43 (31.9)		67 (49.6)		135 (13.3)
	Male		14 (25.0)		13 (23.2)		29 (51.8)	
	Female		11 (13.9)		30 (38.0)		38 (48.1)	
70–79		13 (12.6)		40 (38.8)		50 (48.5)		103 (10.2)
	Male		3 (7.7)		13 (33.3)		23 (59.0)	
	Female		10 (15.6)		27 (42.2)		27 (42.2)	
80<		7 (14.0)		12 (24.0)		31 (62.0)		50 (4.9)
	Male		0 (0)		3 (23.1)		10 (76.9)	
	Female		7 (18.9)		9 (24.3)		21 (56.8)	
	Total	187 (18.4)		346 (34.1)		481 (47.4)		1014 (100)

**Table 2:** Portion of tongue cleaning using a toothbrush.

Portion	Gender	Center^∗^	Entire^∗∗^	Others^∗∗∗^	Total (%)
		*n* (%)	*n* (%)	*n* (%)	
	Male	62 (42.2)	81 (55.1)	4 (2.7)	147 (27.6)
	Female	180 (46.6)	193 (50.0)	13 (3.4)	386 (72.4)

		242 (45.4)	274 (51.4)	17 (3.2)	533 (100)

^∗^The center of the dorsum of the tongue. ^∗∗^The entire tongue. ^∗∗∗^The others.

**Table 3:** Purposes of tongue cleaning using a toothbrush.

Purpose	Gender	To remove tongue stain	To remove tongue coating	To manage halitosis
*n* (%)		*n* (%)	*n* (%)
	Male	95 (64.6)	54 (36.7)	64 (43.5)
	Female	251 (65.0)	138 (35.8)	176 (45.6)
	Total (%)	346 (64.9)	192 (36.0)	240 (45.0)

## Data Availability

The data used to support the findings of this study are available from the corresponding author upon request.
